# Zebra skin odor repels the savannah tsetse fly, *Glossina pallidipes* (Diptera: Glossinidae)

**DOI:** 10.1371/journal.pntd.0007460

**Published:** 2019-06-10

**Authors:** Olabimpe Y. Olaide, David P. Tchouassi, Abdullahi A. Yusuf, Christian W. W. Pirk, Daniel K. Masiga, Rajinder K. Saini, Baldwyn Torto

**Affiliations:** 1 International Centre of Insect Physiology and Ecology (*icipe*), Nairobi, Kenya; 2 Department of Zoology and Entomology, University of Pretoria, Hatfield, South Africa; University of Cincinnati, UNITED STATES

## Abstract

**Background:**

African trypanosomosis, primarily transmitted by tsetse flies, remains a serious public health and economic challenge in sub-Saharan Africa. Interventions employing natural repellents from non-preferred hosts of tsetse flies represent a promising management approach. Although zebras have been identified as non-preferred hosts of tsetse flies, the basis for this repellency is poorly understood. We hypothesized that zebra skin odors contribute to their avoidance by tsetse flies.

**Methodology/Principal findings:**

We evaluated the effect of crude zebra skin odors on catches of wild savannah tsetse flies (*Glossina pallidipes* Austen, 1903) using unbaited Ngu traps compared to the traps baited with two known tsetse fly management chemicals; a repellent blend derived from waterbuck odor, WRC (comprising geranylacetone, guaiacol, pentanoic acid and δ-octalactone), and an attractant comprising cow urine and acetone, in a series of Latin square-designed experiments. Coupled gas chromatography-electroantennographic detection (GC/EAD) and GC-mass spectrometry (GC/MS) analyses of zebra skin odors identified seven electrophysiologically-active components; 6-methyl-5-hepten-2-one, acetophenone, geranylacetone, heptanal, octanal, nonanal and decanal, which were tested in blends and singly for repellency to tsetse flies when combined with Ngu traps baited with cow urine and acetone in field trials. The crude zebra skin odors and a seven-component blend of the EAD-active components, formulated in their natural ratio of occurrence in zebra skin odor, significantly reduced catches of *G*. *pallidipes*by 66.7% and 48.9% respectively, and compared favorably with the repellency of WRC (58.1%– 59.2%). Repellency of the seven-component blend was attributed to the presence of the three ketones 6-methyl-5-hepten-2-one, acetophenone and geranylacetone, which when in a blend caused a 62.7% reduction in trap catch of *G*. *pallidipes*.

**Conclusions/Significance:**

Our findings reveal fundamental insights into tsetse fly ecology and the allomonal effect of zebra skin odor, and potential integration of the three-component ketone blend into the management toolkit for tsetse and African trypanosomosis control.

## Author summary

The use of repellents from non-preferred hosts represents an innovative approach to control animal African trypanosomosis by limiting contact between tsetse flies and livestock. Although zebras are non-preferred hosts, the possible chemical basis of their avoidance by tsetse flies is unknown. We hypothesized that certain chemical components of zebra skin odor play a role in their avoidance. We tested this hypothesis by screening crude zebra skin odor for repellency, identifying the chemicals detected by tsetse flies (*G*. *pallidipes*) in the skin odor matrix of zebra, and establishing the specific chemicals eliciting repellency in field trials. We identified a three-component blend as contributing to the repellency of the crude zebra skin odor. Repellency of the crude zebra skin odor and the three-component repellent blend was comparable to the known tsetse repellent, WRC (waterbuck repellent compounds), derived from waterbuck skin odor. Our study shows that odors play a role in the avoidance behavior of tsetse flies to zebras. The three-component repellent blend identified in zebra skin odor provides a new effective tool in the management of tsetse flies and trypanosomosis.

## Introduction

Tsetse flies (*Glossina sp*.) feed exclusively on blood and are the sole cyclical vectors of the trypanosome parasites that cause African trypanosomosis, a neglected tropical disease [1–3]. Two forms of the disease exist in sub-Saharan Africa, both of which are major constraints to development: Human African Trypanosomosis (HAT) or sleeping sickness, affecting humans, and Animal African Trypanosomosis (AAT) or nagana, affecting livestock, especially cattle [3,4]. While successes have been achieved in recent times to control HAT, the livestock disease is still a huge burden [2] with devastating economic consequences [2,3,5]. Particularly, nagana discourages sustainable agriculture and accounts for at least 3 million cattle deaths annually [4], leading to USD 4.75 billion annual losses in crop and livestock production [3,6]. There is, therefore, an urgent need for enhanced and concerted efforts towards its control.

Control of AAT by chemotherapy has not been sustainable due to widespread and increasing resistance of trypanosomes to existing drugs [2,7], toxicity, relatively high cost and the use of counterfeit or sub-standard drugs in some areas [7,8]. Chemotherapy has also been jeopardized by the presence of wildlife trypanosome reservoirs which effectively maintains the transmission cycle that includes livestock [9,10]. Furthermore, there are no vaccines against any trypanosome pathogen, owing to their complex mechanism of antigenic variation [2,11]. Finally, the use of trypanotolerant cattle is not effective because these breeds are limited in geographical distribution and can lose trypanotolerance when under heavy tsetse densities [6]. Given these challenges, tsetse control efforts constitute a cornerstone in disease suppression and eradication efforts.

Several vector control methods are available for disease management [1,3]. However, strategies exploiting visual and chemical preferences of tsetse flies in traps and targets are the most cost-effective and are particularly promising [1,3]. Adult tsetse flies use a combination of odor and visual cues for host location, the latter being more important for landing [12,13]. By exploiting this host-seeking behavior, blue and black-coloured traps and targets combined with host attractants have been developed to suppress tsetse population by over 90% [3,13]. Here, the blue color of the traps and targets attracts tsetse flies, the black panel triggers landing responses, and the odor cues lure them beyond the vicinity of the trap [13]. For instance, in the Ngu tsetse trap, a blue and black paneled trap for *Glossina pallidipes* patented by *icipe* [14,15], cow urine and acetone are combined with the visual trap as odor cues to enhance trap attractiveness [14]. However, the application of traps and targets is limited to small defined areas [3] and offers no protection to freely-grazing livestock. More versatile vector control strategies addressing these loopholes are warranted for an effective animal African trypanosomosis control.

Innovative mobile tools amenable to African nomadic pastoralists to protect their livestock from tsetse bite and trypanosomosis infection has led to the development of the tsetse repellent technology [14,16]. Certain vertebrates such as waterbuck, zebra, wildebeest and impala are abundant in tsetse habitat but not preferred for blood meals [17–20]. This phenomenon has been strongly linked to allomones emanating from the skin of the non-preferred vertebrate hosts [21] and has been exploited in repellent technology. For instance, potent tsetse repellents have been identified from waterbuck by elucidating the odor basis of the avoidance of this bovid by tsetse flies [22,23]. These have further been harnessed into the development of a four-component tsetse repellent blend comprising geranylacetone, guaiacol, pentanoic acid and δ-octalactone (herein referred to as waterbuck repellent compounds; WRC). This blend is now in use as a tsetse repellent collar to protect livestock [16]. Besides its excellent efficacy, the tsetse repellent technology has been reported to be cost effective compared to available trypanocides [16].

Investigating other non-preferred vertebrate hosts could reveal cheaper, fewer-component repellent blend for tsetse flies with the same or superior potency as the known WRC [16]. Zebras constitute important non-preferred hosts of tsetse flies [17–19]. Previous studies have argued that the striped coats of zebra might play a role in their avoidance by tsetse flies [24,25]. However, the allomonal basis for this avoidance is not understood. Therefore, we tested the hypothesis that much like the waterbuck, zebra skin volatiles contribute to this avoidance behavior of the tsetse fly. Using field trials and chemical analyses, we show that zebra skin volatiles contribute to repellency in the savannah tsetse fly species (*G*. *pallidipes*) and we identify the compounds responsible.

## Methods

### Study sites

**Fig 1:**
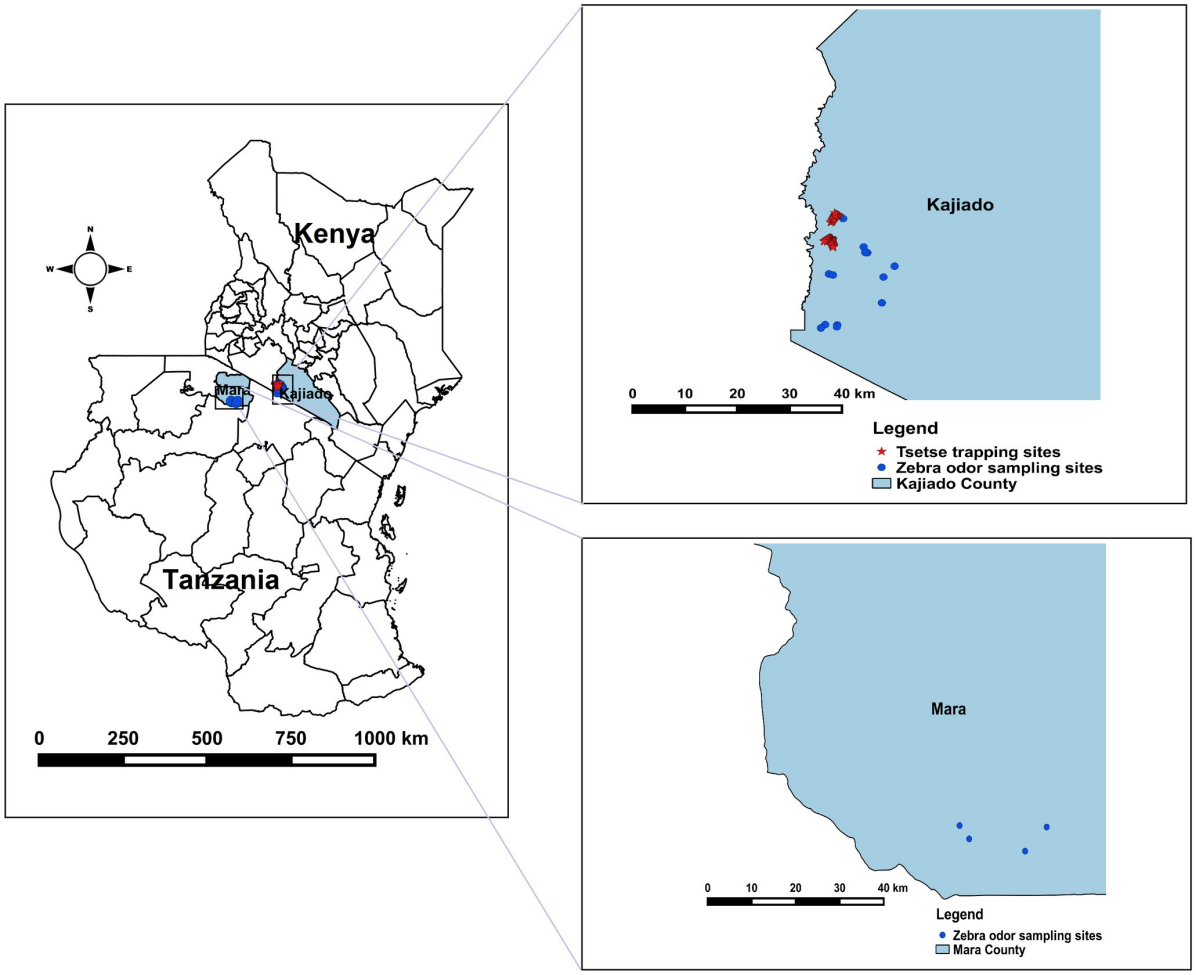
Map of Kenya and Tanzania showing the zebra skin odor sampling and tsetse trapping sites. The maps were designed using QGIS 2.8.3 (http://www.qgis.org/en/site/forusers/download.html), which is an open source software. The coordinates of zebra odor sampling sites and tsetse trapping sites were obtained in the field using a GPS gadget (garmin etrex 20, https://buy.garmin.com/en-US/US/p/518046). The shape files (Kenya, Tanzania) were downloaded from DIVA GIS (http://www.diva-gis.org/gdata), also an open source.

This study was carried out between October 2016 and August 2018. For field evaluations, skin odors of zebra were collected in Nguruman (Kajiado County, Kenya) (Fig 1). Similar zebra odor sampling was conducted in Serengeti (Mara County, Tanzania) but only used for laboratory analysis. Nguruman maintains an abundant community of savannah tsetse flies (*G*. *pallidipes* Austen) and a forest species (*G*. *longipennis*) [22,26] whereas, in Serengeti, two savannah tsetse fly species (*G*. *pallidipes* and *G*. *swynnertoni*) are present [17]. Both areas have similar vegetation cover, ranging from open savannah grasslands to dense woodlands [17,26] suitable for the savannah species of tsetse flies [27] which was the focus of the present study. The choice of the study sites was informed by the co-existence of both zebra and tsetse flies in the same natural habitat. In addition to zebra, both areas have diverse community of wildlife species such as giraffe, wildebeest, elephant, antelope, buffalo, warthog, gazelle, waterbuck, bushbuck and non-human primates.

### Zebra immobilization, skin odor collection and processing

Adult plains zebras (*Eguus quagga*) were accessed *in situ* in Nguruman and Serengeti with the help of the Kenyan Wildlife Service (KWS) and the Tanzanian Wildlife Research Institute (TAWIRI) personnel, respectively. Zebras were immobilized and anaesthetized with 0.015mg/ kg body weight opiate etorphine hydrochloride (Captivon 98, Wildlife Pharmaceuticals Ltd, South Africa) in combination with 0.20mg/kg body weight azaperone (Kyron Laboratories, South Africa) [28]. All zebras were positioned on lateral recumbency to prevent respiratory complications and facilitate efficient working and sampling. Skin odors were collected from fourteen plains zebras including seven males and seven females by rubbing Soxhlet-extracted (with dichloromethane) and oven-dried cotton materials (23 cm x 23 cm, Lux Premium, Bidhannagar, West Bengal, India) on the belly and upper parts of the front legs where most tsetse flies are known to feed [29] for 10–12 min [30]. The zebra anaesthesia was reversed with intravenous injection of opioid antagonist diprenorphine (Activon, Wildlife Pharmaceuticals Ltd, South Africa) at a dosage rate of 0.045mg/kg body weight [28]. All animals recovered well with no complications after antagonist administration. To minimize contaminations with human odor and zebra fecal droppings, latex-gloved hands were used to collect skin odors and the rear legs, anal region and genitals of the animals were carefully avoided [31]. Collected odor samples were immediately wrapped in at least four layers of aluminum foil, placed in separate ziplock plastic bags and kept in a cool box underlaid with dry ice for use in field trapping experiments and laboratory analyses.

### Traps

The Ngu tsetse trap (100% polyester, Vestergaard Frandsen, Lausanne, Switzerland) was used throughout this study. The traps were placed about 200 m apart and the odor dispensers were placed at the base of each trap approximately 30 cm downwind according to the standard procedures adopted for trapping tsetse flies [12,22].

### Field evaluation of the crude skin odors of zebra

We monitored trap catches of tsetse flies (*G*. *pallidipes*) in a series of randomized 3 x 3 Latin square designed experiments [12,22] with treatments comprising i) crude zebra skin odor, ii) WRC, a known tsetse repellent as negative control and iii) a combination of cow urine and acetone, known attractants, as positive control. Each treatment was replicated 30 times. Five pieces of cotton materials with crude skin odors of zebra were placed next to the Ngu tsetse trap in cylindrical canisters (diameter 9.5 cm, height 22.5 cm) fabricated from a stainless-steel wire mesh (1 mm x 1 mm x 1 mm sq mesh holes). WRC sachets (2 sachets of 4.5 ml per trap) were made of polyethylene (0.15 mm thickness, 50 cm^2^ surface area) folded and double-sealed into a tetrahedron shape [22]. Trap catches were recorded daily (between 1100 and 1300 hr, after the morning activity period which is usually between 0900 and 1100 hr) [32] to ensure captures of *G*. *pallidipes* both during their morning and afternoon activity periods. Daily trap catches were sorted by species and sex. The treatments were rotated daily according to the Latin square design and fresh zebra skin odors were used for each 24 h trapping period.

### Zebra skin odor trapping

Aliquot samples of zebra skin odors were trapped on site on to adsorbent filters (Carbopak B 3.5” with 30 mg ± 5 mg, Sigma Scientific, Gainesville, Florida, USA) in a volatile entrainment system using a battery-powered field pump (assembled at the USDA/CMAVE, Gainesville, Florida, USA) [31]. Headspace odors from the cotton materials containing freshly collected zebra skin odor were trapped on to the adsorbent filter for 12 hr by the field pump preset to supply charcoal-filtered clean air at a flow rate of 348 ml/min. Adsorbent filters containing trapped odors were tightly sealed with Teflon tape, wrapped in aluminum foil in a cool box underlaid with dry ice and later transported to the laboratory in *icipe*, Duduville campus. Trapped skin odors were eluted from each adsorbent filter with 200 μl dichloromethane (≥ 99.9% Sigma-Aldrich, St Louis, Missouri, USA) and concentrated to 100 μl under a very gentle stream of charcoal-filtered nitrogen. Eluted skin odor extracts were kept at -80 °C until use.

### Analysis of volatiles

Gas chromatography-electroantennographic detection (GC/EAD) analysis was carried out on a HP 5890 GC model fitted with a non-polar capillary column HP-1 stationary phase (30 m x 0.2 mm x 0.2 μm film thickness) and a flame ionization detector (280 °C) operated on a splitless injector mode (220 °C). Three three-day-old adult females *G*. *pallidipes*, reared in the laboratory under 12L:12D photoperiod, 25 ± 2 °C and 70 ± 5% relative humidity [33], were used. Individual insect that was fed two days earlier was immobilized on ice and the whole insect was mounted on a microscope stage between two capillary glass tubes filled with Ringer solution [34,35]. The tip of the insect antenna was gently inserted into the capillary tube on the recording electrode and to complete the circuit, the capillary tube at the reference electrode, grounded by an Ag-AgCl wire, was inserted to the base of the antenna. The column oven was held at 35 °C for 5 min, thereafter programmed to increase at 10 °C/min to 280 °C and maintained at this temperature for 10 min. An aliquot (2 μl) of the crude zebra skin odor extract was injected into the GC and components were separated on the column under the temperature programming mode with nitrogen as a carrier gas at 1.2 ml/min flow rate. Upon exit, nitrogen make-up gas was added to the column effluent, split 1:1 and delivered to the FID and the antenna of the mounted tsetse fly, via a stainless-steel delivery tube (5 mm ID), for simultaneous detection by the FID and EAD. The recordings were later analyzed with GC/EAD 2000 software (Syntech, Hilversum, the Netherlands). Commercially purchased authentic standards of identified EAD-active compounds were also analyzed under similar conditions as the crude odor samples.

Samples (1 μl) were analyzed on an Agilent GC/MS (Agilent technologies 7890A series). The GC was fitted with an autosampler, a split-splitless injection port (200 °C), an HP-5 Agilent fused silica capillary column (30 m length x 0.2 mm id x 0.22 μm film thickness), an Agilent technologies 5975C EIMS (electron energy 69.922 eV) triple axis mass selective detector (MSD), and an Agilent ChemStation data system [31]. The injection port ran on a splitless mode and the column oven ran on a temperature programmed mode as in the EAD but with helium as the carrier gas (1.2 ml/min flow rate, 8.8271 psi head pressure). After tentative identification of the EAD-active compounds in zebra skin odor extract, a final validation of identities was conducted using commercial standards (1 μl in 4000 μl dicholoromethane) made in into blends (aldehydes, ketones) in the EAD, and by comparing their GC retention indices and MS fragmentation patterns. Further, an external quantification of the identified EAD-active compounds was achieved using calibration curves prepared for each class of compounds over five different known concentrations within their expected range in the zebra odor extract. Nonanal was selected for the aldehydes, geranylacetone for the aliphatic ketones and acetophenone for the benzenoid ketone. These calibration curves were obtained using the peak area of each selected compound over three replicate runs for each concentration. Using the resulting linear equation from the calibration curves, the concentrations (ng/μl) and their natural ratio in the zebra odor were estimated.

### Chemicals

The chemicals used included: 6-methyl-5-hepten-2-one (Aldrich, 99%), acetophenone (Sigma-Aldrich, ≥ 99%); geranylacetone (Aldrich, 65% geranylacetone and 35% nerylacetone), heptanal, octanal, nonanal and decanal (Aldrich, 95%).

### Field evaluation of identified EAD-active compounds in zebra skin odor

To evaluate their effects on trap catches of *G*. *pallidipes*, seven identified EAD-active compounds—heptanal, 6-methyl-5-hepten-2-one, octanal, acetophenone, nonanal, decanal and geranylacetone—were tested in blends and singly in a series of Latin square-designed field experiments [12,22]. This field evaluation was carried out in three experiments described below. The compounds were used neat and to prevent oxidation, 10% antioxidant (2,6-di-tert-butyl-4-methylphenol, Aldrich, Gillingham—Dorset, UK) was added to each aldehyde before use [31]. Blends of compounds were constituted to mimic their natural ratio of occurrence in zebra skin odors (S1 Table) and dispensed from polyethylene sachets (0.15 mm thickness, 50 cm^2^ surface area) folded and double-sealed into a tetrahedron shape as used for WRC. Two 4.5 ml sachets of individual compounds and blends, pre-informed as the optimum repellent doses in initial trials with one, two and three sachets, were used per trap, except for the seven-component blend (Blend Z) in which three sachets gave the optimum repellency (S2 Table).

### Experiment I: Evaluating the effect of a blend of all the EAD-active compounds

We evaluated the effect of a seven-component blend of the identified EAD-active compounds on field catches of *G*. *pallidipes* in Ngu traps combined with attractants (cow urine and acetone) in a 3 x 3 randomized Latin square designed experiments replicated 10 times. The treatments evaluated were: (i) attractant-baited (cow urine and acetone) Ngu trap (positive control), (ii) attractant-baited trap with WRC (negative control), and (iii) attractant-baited trap with Blend Z (7-component blend of EAD-active compounds mimicking natural zebra skin odor).

### Experiment II: Evaluating the repellent effect of each identified compound

Here, we assessed the relative contribution of each EAD-active compound to the observed repellency in field trials. We used two sachets each containing 4.5 ml of the individual compounds, predetermined as optimum repellent dose based on trap catches as described earlier (S2 Table). For the aldehydes (heptanal, octanal, nonanal, decanal), a 6 x 6 Latin square design was used, and each treatment tested in 12 replicate trials comprising: (i) attractant-baited (cow urine and acetone) Ngu trap (positive control); (ii) attractant-baited trap with WRC (negative control); (iii-vi) attractant-baited trap with each of the four aldehydes, separately. For the ketones (6-methyl-5-hepten-2-one, acetophenone, geranylacetone), a 5 x 5 Latin square design was used and each treatment, listed as follows, had 10 replicates: (i) attractant-baited (cow urine and acetone) Ngu trap (positive control); (ii) attractant-baited trap with WRC (negative control); (iii-v) attractant-baited trap with each of the three ketones, singly.

### Experiment III. Evaluating the repellent effect of identified compounds by class

In this experiment, blends of all seven EAD-active compounds, all four aldehydes and all three ketones were compared using a 5 x 5 Latin square with components tested as follows in 10 replicate trials: (i) attractant-baited (cow urine and acetone) Ngu trap (positive control); (ii) attractant-baited trap with WRC (negative control); (iii) attractant-baited trap with blend A (blend of four aldehydes); (iv) attractant-baited trap with blend K (blend of three ketones); (v) attractant-baited trap with blend Z (blend of all seven EAD-active compounds in zebra skin odor).

Additionally, we explored the possibility of having a 2-component tsetse repellent blend of ketones (using acetophenone and geranylacetone, which showed higher repellency than 6-methyl-5-hepten-2-one when tested individually) instead of a 3-component blend. The 3-component repellent blend was also compared with trap alone (unbaited Ngu trap). The experiment followed a 5 x 5 Latin square design having the following components evaluated in 20 replicates each: (i) attractant-baited (cow urine and acetone) Ngu trap (positive control); (ii) trap alone; (iii) attractant-baited trap with WRC (negative control); (iv) attractant-baited trap with Blend K (three-component blend of ketones); (v) attractant-baited trap with 2C Blend K (two-component blend of ketones—acetophenone and geranylacetone). All the blends tested above (blend A, blend K, blend Z, 2C blend K) were constituted to mimic the natural ratios of their occurrence in zebra skin odor (S1 Table). The dose used for each blend (number of sachets) was the optimum repellent dose determined in preliminary trials (S2 Table).

### Data analysis

Daily trap catches of *G*. *pallidipes* for each treatment were analyzed in R (version R i386 3.2.3) using a generalized linear model with negative binomial error structure with abundance as response variable and treatment, day and site as predictor variables. Mean catches and standard error for each treatment were calculated. Means were separated using ‘lsmeans ()’ function, embedded in Least-Squares Means (lsmeans) R package, with Tukey adjustment for post hoc comparison. Further, the percentage reduction in catches for each treatment compared to the control was calculated using the mean catches. The higher the catch reduction, the more the treatment is avoided by savannah tsetse flies (*G*. *pallidipes*) and the better it is as a repellent for the fly. For all statistical tests α was set at 0.05.

### Ethics statement

The use of zebra in this study was approved by the KWS (permit number: KWS/BRM/5001) the TAWIRI, the Tanzania National Park (TANAPA) and the Commission for Science and Technology (COSTECH) (COSTECH permit number: 2016-223-NA-2016-96). In addition, consent was sought from community elders in Nguruman before traps were set in the forest.

## Results

### Effects of crude zebra skin odor on field trap catches of *G*. *pallidipes*

**Fig 2:**
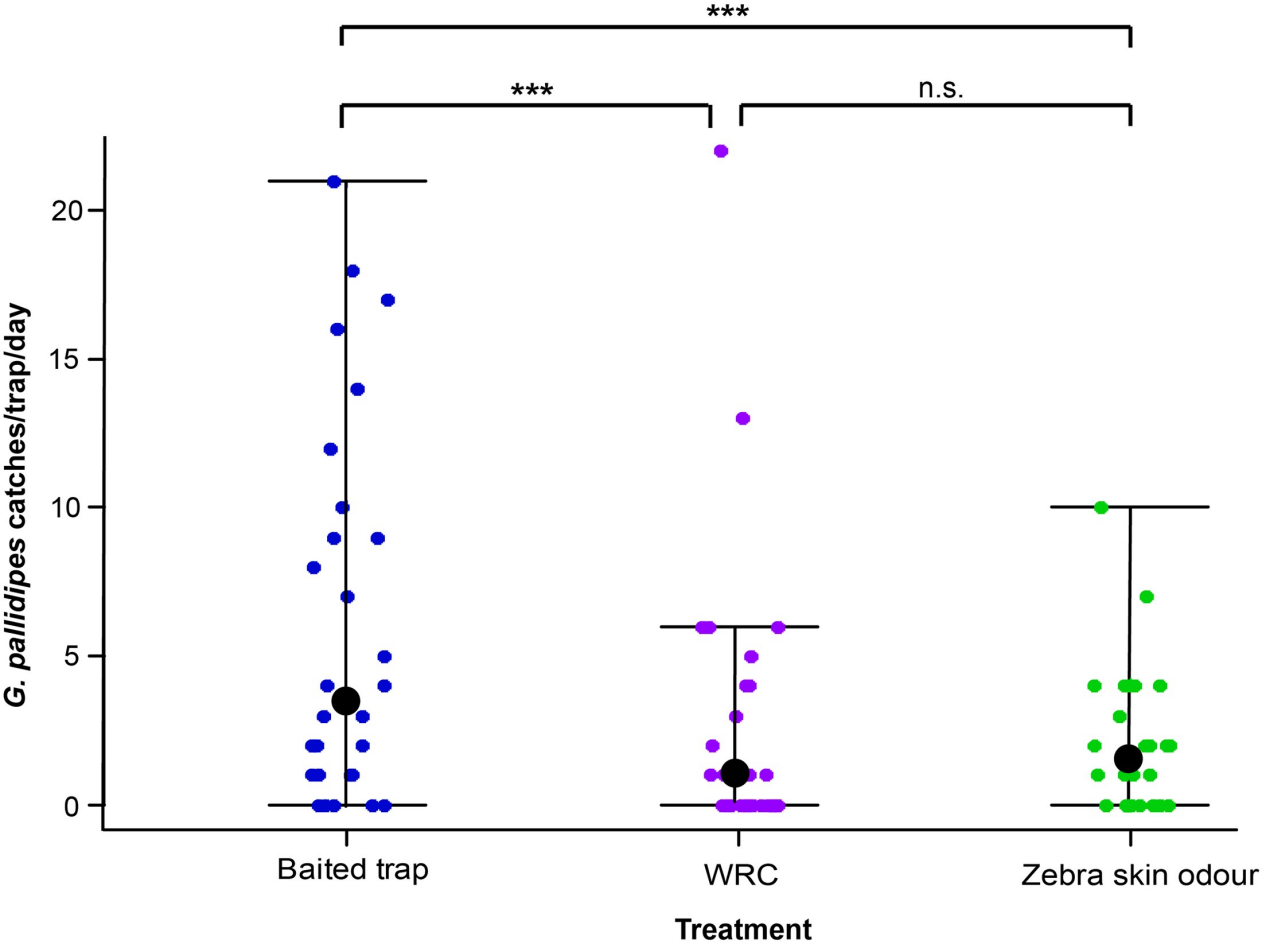
Daily catch of *G*. *pallidipes* in Ngu traps combined with crude zebra skin odor in 30 replicate trials. Dot plots showing the daily trap catches of *G*. *pallidipes*. The boundaries of the dot plot whiskers represent the minimum and maximum of all the count data. Dots outside the boundaries are outliers and the black dots represent the median number of catches. Experiment was carried out in 3 x 3 Latin square designs. baited trap, positive control; WRC, negative control; *** = p < 0.001; n.s. = no significant difference.

A total of 326 *G*. *pallidipes* were caught (82.2% female, 17.8% male). Crude skin odors of zebra significantly reduced trap catch of *G*. *pallidipes* (66.7% catch reduction, IRR = 0.33, p < 0.001) when compared to a baited trap (cow urine and acetone, positive control). However, its repellent effect was not significantly different from WRC (58.1% catch reduction, IRR = 0.42, p < 0.001) (Fig 2). In addition to *G*. *pallidipes*, *G*. *longipennis* (a forest species of tsetse flies) was trapped. Fifty-one *G*. *longipennis* were caught in the baited trap and only four were caught in the trap containing zebra odor, translating to a 92.2% catch reduction (IRR = 0.08, p < 0.001). Similarly, ten *G*. *longipennis* were caught in the trap containing WRC (catch reduction = 80.4%, IRR = 0.20, p < 0.001).

### Electrophysiological responses of *G*. *pallidipes* antennae to zebra skin odor extract

**Fig 3:**
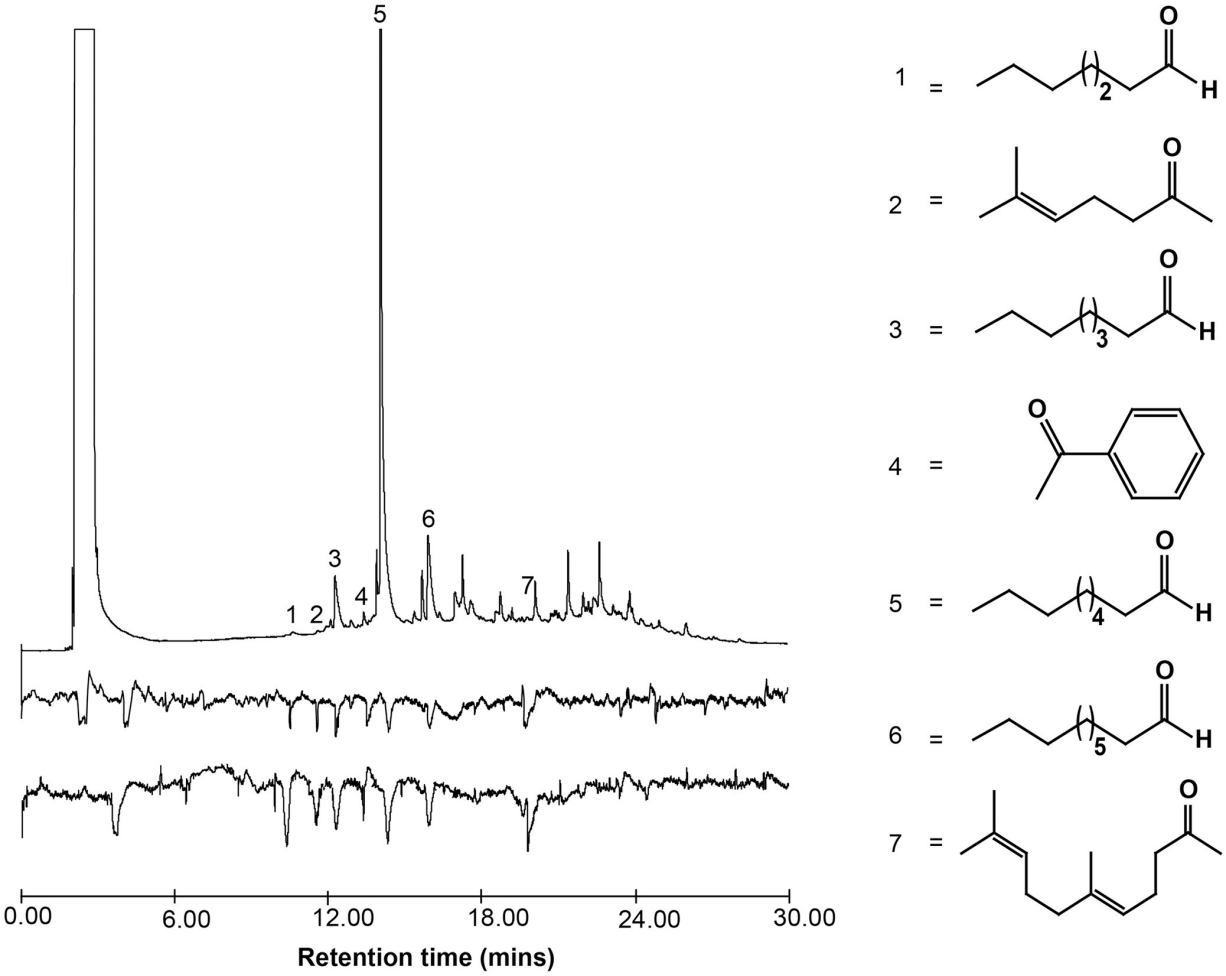
Representative GC/EAD profiles showing antennal responses of *G*. *pallidipes* to crude skin odors of zebra. Upper trace is the GC/FID and lower traces represent EAD responses. Chemical structures of the identified EAD-active compounds are shown. 1, heptanal; 2, 6-methyl-5-hepten-2-one; 3, octanal; 4, acetophenone; 5, nonanal; 6, decanal; and 7, geranylacetone. The EAD runs were scaled to 10mV/div.

In GC/EAD analysis, the antennae of three individual female *G*. *pallidipes* consistently detected seven components which were present in small amounts in the extract (Fig 3). These components were identified as the aldehydes heptanal, octanal, nonanal, decanal and ketones 6-methyl-5-hepten-2-one, geranylacetone, and acetophenone (Table 1). The identities of the EAD-active components were further confirmed using commercially-purchased standards, however, the antennal response to some of the synthetic compounds is reduced as compared to components detected in the zebra extract (S1 Fig). Notably, the response of the olfactory sensory neuron (OSN) was higher for the initial stimulations but this response declined with stimulations from the latter eluting ketones (S1 Fig).

**Table 1:** Kovats retention index and amount (± SEM) of identified EAD-active compounds in zebra skin odor (n = 3).

Compound name	Retention time (t_R_)	Kovats retention index (*I*)	Amount (ng/μl)
Heptanal	9.22 ± 0.04	907	35.53 ± 1.89
6-methyl-5-hepten-2-one	11.03 ± 0.00	993	48.01 ± 0.77
Octanal	11.34 ± 0.02	1009	152.18 ± 34.92
Acetophenone	12.54 ± 0.04	1076	80.11 ± 12.87
Nonanal	13.18 ± 0.01	1114	837.65 ± 158.36
Decanal	14.76 ± 0.01	1214	277.58 ± 78.93
Geranylacetone	18.19 ± 0.03	1465	65.38 ± 7.93

Samples were analyzed on a HP-5 Agilent fused silica capillary column. n, total number of replicates. Amounts were calculated from calibration equations generated from five known concentrations of commercial standards, within their range of occurrence in the zebra skin odor extract.

### Effects of the EAD-active components of zebra skin odor on field catches of *G*. *pallidipes*

**Fig 4:**
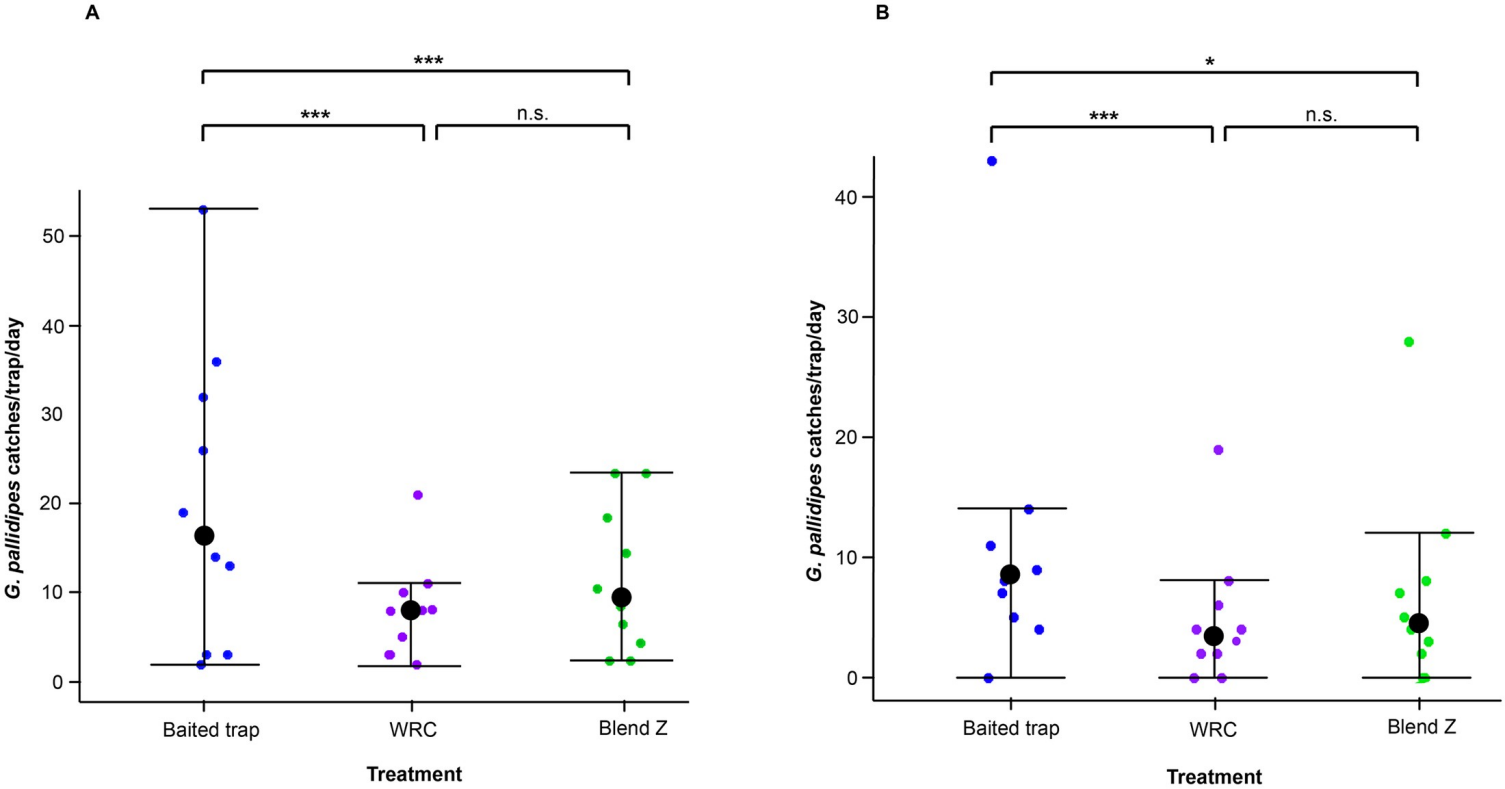
Daily catches of *G*. *pallidipes* in baited trap combined with Blend Z in ten replicate trials. Dot plots showing the daily trap catches of *G*. *pallidipes* females (A) and males (B). The boundaries of the dot plot whiskers represent the minimum and maximum of all the count data. The dots outside the boundaries are outliers. Black dots represent the median number of catches. Experiment followed a 3 x 3 Latin square design. Baited trap, positive control; WRC, negative control; Blend Z, 7-component blend of EAD-active compounds mimicking zebra skin odor. * = p < 0.05; *** = p < 0.001; n.s. = no significant difference.

In the experiment evaluating the seven-component blend of all EAD-active compounds on field catches in baited (cow urine and acetone) traps, a total of 597 *G*. *pallidipes* were collected (62.0% female, 38.0% male). As observed for crude zebra skin odor, the 7-component blend resulted in significant reduction (p < 0.001) in the catch of *G*. *pallidipes* [catch index = 0.42, 95% confidence interval, CI (0.28–0.61)] compared to the baited trap alone (positive control). The reduction in catches was recorded for both males and females of *G*. *pallidipes* which were not significantly different from that exhibited by the WRC (Fig 4).

In the experiment examining the effects of the individual compounds, a total of 2,693 *G*. *pallidipes* were caught (67.7% female, 32.3% male). The individual aldehydes (heptanal, octanal, nonanal, decanal) had no significant effect on *G*. *pallidipes* catches in baited traps (positive control). However, for the individual ketones, acetophenone (CI = 0.57) and geranylacetone (CI = 0.57), demonstrated significant reduction in catches (Table 2).

**Table 2:** Mean catches (± SEM) and estimated catch indices of pooled males and females *G*. *pallidipes* in cow urine and acetone-baited Ngu traps combined with individual compounds.

Aldehydes (n = 12)					Ketones (n = 10)				
Treatment	Mean catches*	% CR	Estimate	Catch index	Treatment	Mean catches*	% CR	Estimate	Catch index
Baited trap	28.17 ± 5.65 ^b^	-	-	-	Baited trap	31.40 ± 7.72^b^	-	-	-
Baited trap + WRC	6.58 ± 2.21^a^	76.6	-1.61	0.20	Baited trap + WRC	11.40 ± 3.77^a^	63.7	-1.09	0.34
Baited trap + 1	20.67 ± 5.02^b^	26.6	-0.35	0.71	Baited trap + 2	28.10 ± 8.60^ab^	10.5	-0.28	0.76
Baited trap + 3	33.17 ± 15.47^b^	-17.8	-0.13	0.88	Baited trap + 4	17.10 ± 3.93^ab^	45.5	-0.55	0.57
Baited trap + 5	25.75 ± 7.83^b^	8.6	-0.25	0.78	Baited trap + 7	21.30 ± 5.98^ab^	32.2	-0.57	0.57
Baited trap + 6	19.00 ± 2.74^b^	32.5	-0.34	0.71	-	-	-	-	-

Experiments were carried out in 6 x 6 and 5 x 5 Latin square designs for the aldehydes and ketones, respectively. Means with the same letter, within the same column, are not significantly different. n, total number of replicates for each treatment; CR, catch reduction. 1, heptanal; 2, 6-methyl-5-hepten-2-one; 3, octanal; 4, acetophenone; 5, nonanal; 6, decanal; and 7, geranylacetone. Baited trap, positive control; WRC, negative control.

A total of 996 *G*. *pallidipes* were caught (62.6% female, 37.4% male) in the experiment comparing the identified compounds grouped by class. Baited Ngu traps combined with the four-component blend of the aldehydes (blend A) did not significantly impact catch reduction of *G*. *pallidipes* compared with the baited trap alone (positive control). On the other hand, baited traps combined with the three-component blend of ketones (blend K) significantly reduced the catch of *G*. *pallidipes* (Catch index 0.39, p < 0.001), which compared favorably with the trap catch recorded for the seven-component blend and WRC (Table 3).

**Table 3:** Mean trap catches (SEM) and catch indices of *G*. *pallidipes* in cow urine and acetone-baited Ngu traps combined with different blends (n = 10).

	Female				Male			
Treatment	Trap catches*	% CR	Estimate	Catch index (95%)	Trap catches*	% CR	Estimate	Catch index (95%)
Baited trap	20.1 ± 5.28^c^	-	-	-	11 ± 3.76^c^	-	-	-
Baited trap + WRC	7.9b ± 1.75^a^	60.7	-1.02	0.36 (0.23–0.56)	4.8 ± 1.76^a^	56.4	-1.01	0.37 (0.22–0.6)
Baited trap + Blend A	17.1 ± 5.24^bc^	14.9	-0.34	0.71 (0.47–1.06)	11.2 ± 3.49^bc^	-1.8	-0.14	0.87 (0.56–1.34)
Baited trap + Blend K	8.2 ± 1.91^ab^	59.2	-0.82	0.44 (0.29–0.67)	3.4 ± 0.87^a^	69.1	-1.11	0.33 (0.19–0.55)
Baited trap + Blend Z	9.00 ± 2.56^a^	55.2	-0.99	0.37 (0.24–0.57)	6.9 ± 2.62^ab^	37.3	-0.71	0.49 (0.31–0.79)

Experiment was carried out following a 5 x 5 Latin square design. Means with the same letter, within the same column, are not significantly different. n, total number of replicates for each treatment; CR, catch reduction. Blend A, K, and Z indicates 4-component blend of aldehydes, 3-component blend of ketones and 7-component blend of all EAD-active compounds, respectively, in natural ratios of occurrence in zebra skin odor. Baited trap, positive control; WRC, negative control.

An additional experiment which compared the 3-component blend of ketones above with a 2-component blend (acetophenone and geranylacetone) with baited trap (cow urine and acetone) as positive control, recorded 1,658 *G*. *pallidipes* (72.4% female, 27.6% male). The two-component blend (2C blend K) reduced field catches of *G*. *pallidipes* (IRR 0.59) and the performance was, again, not significantly different from WRC (IRR 0.71). However, blend K (the 3-component ketone blend) performed better than the 2-component blend (IRR 0.42) in repelling *G*. *pallidipes*. Catches of *G*. *pallidipes* in baited traps, combined with either “blend K”, “2C blend K” or “WRC”, were not significantly different from the catches in the trap alone (Table 4).

**Table 4:** Mean catches (± SEM) and catch indices of pooled males and females *G*. *pallidipes* in baited Ngu traps combined with different treatments (n = 20).

Treatment	Trap catches*	% CR	Estimate	Catch index (95% CI)
Baited trap	25.15 ± 5.89^b^	-	-	-
Trap alone	14.25 ± 2.31^ab^	43.3	-0.46	0.63 (0.37–1.06)
Baited trap + WRC	17.85 ± 4.22^ab^	29.0	-0.36	0.70 (0.41–1.20)
Baited trap + Blend K	10.75 ± 2.49^a^	57.3	-0.95	0.39 (0.23–0.65)
Baited trap + 2C Blend K	14.90 ± 2.43^ab^	40.8	-0.38	0.68 (0.41–1.14)

Experiment followed a 5 x 5 Latin square design. Means with the same letter, within the same column, are not significantly different. n, total number of replicates for each treatment; CU + Ac, cow urine + acetone; CR, catch reduction. “Blend K” and “2C Blend K” indicates 3-component blend of ketones and 2-component blend of ketones, respectively, in their natural ratios of occurrence in zebra skin odor. Baited trap, positive control; WRC, negative control.

## Discussion

Exploiting odors of non-preferred vertebrate hosts of tsetse flies for repellents represents a sustainable innovative strategy for the control of both Human and Animal African Trypanosomosis [16]. Here, we established the presence of potent repellents in zebra skin odor for male and female savannah tsetse fly *G*. *pallidipes*. This observed repellency was maintained regardless of gender biases in field captures of *G*. *pallidipes* which may relate to differences in their population due to seasonal variations. This is particularly important since both male and female tsetse flies feed exclusively on blood and can transmit African Trypanosomosis [3]. The observed repellency of crude skin odor of zebra and a 7-component blend of the identified EAD-active compounds simulating their natural ratio of occurrence in zebra skin odor strongly supported our study hypothesis. Further, we formulated and identified a 3-component blend of ketones repellent to *G*. *pallidipes*. Subject to further field evaluation for performance across different seasons and ecologies, this blend could be used to protect livestock, particularly cattle, from tsetse bites and trypanosome infections.

We found that the crude skin odor of zebra was effective in reducing field trap catches of savannah tsetse flies, like the known tsetse repellent, WRC. Previous research has shown that zebras, although present in tsetse habitat, are usually avoided by these flies [17–19]. A previously proposed hypothesis for this avoidance suggests that the polarization effects of the striped pelage of zebras is the driving component of the observed avoidance [24]. However, the stripes of this ungulate is visible to tsetse flies only at a distance of about 5–10 m and beyond this distance, zebras appear uniformly grey to these flies [36]. This indicates that fitness benefits conferred by the stripes on zebra skin against tsetse flies are only within the boundaries of this proximity. Also, tsetse flies utilize odor cues in locating suitable hosts and discriminating potentially unsuitable vertebrates [21]. In this case, the odor cues might complement visual cues in conferring fitness benefits beyond the vicinity of zebras. Our study shows that odor cues from zebra skin contribute to the avoidance behavior of tsetse flies to this equid. This finding is not characteristic of zebras alone. Skin odors of non-preferred vertebrates of hematophagous arthropods are known to contain potent repellents. For example, beagle dogs are avoided by the brown dog tick [37]. Similarly, chickens are avoided by *Anopheles* mosquitoes [38], and waterbucks are avoided by tsetse flies [23]. The skin odors of each of the aforementioned contain repellents for these blood feeding arthropods [23,37,38] and are being exploited for their potential to control these flies. Our study does not rule out the importance of visual cues in the avoidance behavior of tsetse flies to zebras but shows the contribution of chemical cues from the skin odor. Future studies can focus on exploiting possible synergy between visual and odor cues in this avoidance behavior and its possible integration as a novel strategy in protecting livestock hosts from tsetse bites. More research can also be conducted to include other sources of odors including zebra breath, urine and dung.

Our current study identified key EAD-active aldehyde and ketone components of zebra skin odor extract, which were present at different concentrations and showed varying magnitudes in *G*. *pallidipes* antennal responses. The magnitude of antennal responses depends on several factors such as the nature and concentration of the stimulus, number and strength of previous stimulations, insect species and the quality of the antennae [34]. Furthermore, the distribution of the olfactory sensilla for some compounds are localized [39], and antennal response could be affected by the mounting technique. This could contribute to the lower response to heptanal compared to the latter aldehyde stimulations. Also, adaptation due to repeated stimulation of the olfactory sensory neurons (OSNs) as the individual compounds elute from the GC column is possible. As shown both in insect and mammalian olfactory sensory neurons, repeated and longer stimulation with high concentration induce OSN adaptation [40–42]. This adaptation due to repeated stimulations and higher concentrations could explain the reduction in responses to the latter-eluting compounds. For example, as noted for the response to geranylacetone when the commercially-purchased ketones were used as stimulus compared to the natural zebra skin odor extract. The reduced EAD response for subsequent GC stimulation could also enable us to hypothesis that the ketones most probably are detected by the same receptor [43]. However, electroantennographic detection by an insect does not necessarily translate to behavioral responses and such data are better used as a qualitative indicator of antennal responses [35]. For instance, in the moth *Manduca sexta*, odor detection by females did not translate to increased behavioral response of the females compared to males [44]. Therefore, a detailed behavioral assay with the identified EAD-active compounds is required, as conducted in our study.

The identifed EAD-active ketones (geranylacetone, acetophenone and 6-methyl-5-hepten-2-one) and the aldehydes (heptanal, octanal, nonanal and decanal) are constituents of skin odors of certain vertebrates, and different blood feeding insects will show variant behavioral responses to these odors [22,33,38,45–47]. Geranylacetone is present in waterbuck skin odor [33] and a key contributor to the repellency of WRC [22]. This compound has also been shown to repel effectively several mosquito disease vectors [46]. It could be prudent to investigate the probable utilization of geranylacetone as biomarkers for potentially unsuitable vertebrates for feeding to tsetse and other hematophages. Also, the repellence of acetophenone to tsetse flies has previously been established [47]. The ketone 6-methyl-5-hepten-2-one is a signature component of human odor [48], and has been shown to have either attractive or repellent effects on mosquitoes, depending on the dose and formulation [45,46,49]. In our study, 6-methyl-5-hepten-2-one alone did not impact trap reduction of tsetse flies. However, when combined with acetophenone and geranylacetone, it contributed to the repellency of the 3-component blend by increasing the catch reduction by about 50%. We recorded a more significant repellency when the three ketones were formulated into a blend, than as individual compounds, thus suggesting a combined activity of these chemicals. Also, 6-methyl-5-hepten-2-one could have some combined effects on lactic acid which is present in human skin odor, and was previouly shown to be the major chemical responsible for the avoidance of humans by tsetse flies [50]. The identified EAD-active aldehydes (heptanal, octanal, nonanal, decanal) are commonly found in skin odors of vertebrates, and are attractive to blood feeding insects [31,33,38]. For example, they are present in skin odors of preferred (buffalo, cattle) and non-preferred (waterbuck) vertebrate hosts of tsetse [33]. In previous laboratory experiments, these aldehydes were suggested as attractants for tsetse flies [23]. It was therefore not surprising that the aldehydes had no significant effect on field catches of tsetse flies in cow urine and acetone-baited Ngu traps when used either alone or in blends.

The empirical application of tsetse repellents in disease control has recently been demonstrated [16]. Collars containing repellent chemicals worn by cattle can be used to protect them from tsetse bites and trypanosomosis infection either alone, as these livestock are grazed in tsetse infested areas, or in a push-pull strategy, in which the repellent collars serve as push and a attractant-baited trap/target serve as a dead-end pull. This approach will not only reduce disease incidence and trypanocide use, but also reduce tsetse population over time. This is particularly advantageous as the tsetse repellent technology becomes more efficient as the disease incidence and tsetse population reduce [51]. Repellents for tsetse flies can also be successfully applied in areas where trypanotolerant cattle are in use, and in the control of HAT because of the characteristic low infection rates of *Trypanosoma brucei species* [51]. Key strengths of this approach over the existing ones are its mobility and ease of use which have triggered Kenyan livestock farmers’ interest in embracing the technology in tsetse and trypanosomosis control [16,51]. Like the existing tsetse repellent, WRC, the newly identified repellent blend also contains geranylacetone, but the other components are different. However, unlike the WRC which contains a ketone, an alcohol, an acid and a lactone, the repellent blend identified in our study consists of only ketones and are more likely to maintain their integrity for longer periods of time, although this needs to be established in future studies. More specifically, the blend is made up of three components and may offer a cheaper, yet potent, alternative to WRC. Also, the new repellent blend could have synergistic effect if combined with WRC though this needs to be determined in further studies. This repellent technology could be combined with other tsetse and African trypanosomosis control strategies for their integrated management.

### Conclusion

We investigated the chemical basis of the interactions between zebras and tsetse flies and established that odor contributes to the avoidance behavior of tsetse flies to zebras. Our results show that, like the known tsetse repellent WRC, ketones present in zebra skin odor are largely responsible for the tsetse fly avoidance behavior. The three-component ketone blend can be incorporated into the tsetse repellent technology to protect livestock, particularly cattle, from tsetse bites and trypanosome infections. We recommend multi-site field testing of this newly identified tsetse repellent blend and evaluation of their effect on disease incidence in livestock in tsetse-infested areas. Finally, we recommend future studies to explore a possible interaction between odor and vision in the tsetse-zebra interactions and the comparison of skin odor profiles of zebra with other equids that are relatively more attractive to tsetse flies.

## Supporting information

S1 Table Amount (in grams) of each compound in 4.5 g of each blend reflecting their natural ratios of occurrence in zebra skin odor. (TIF)

S2 Table Mean catches ± SEM in the initial trials to determine the optimum repellent doses for each compound and blend in three replicates trials. Blend A, K, and Z indicate 4-component blend of aldehydes, 3-component blend of ketones and 7-component blend of all EAD-active compounds, respectively, in their natural ratios of occurrence in zebra skin odor. Values underlined represent catches at optimum repellent dose (i.e. dose with the least catch or not significantly different from the dose that did).
(TIF)

S3 Table Release rates ± SEM of individual compounds and blends from polyethylene sachets in three replicates. Blend A, K, Z and 2C Blend K indicate 4-component blend of aldehydes, 3-component blend of ketones, 7-component blend of all EAD-active compounds and 2-component blend of ketones respectively, in their natural ratios of occurrence in zebra skin odor.
(TIF)

S1 Fig GC/EAD profiles showing antennal responses of *G*. *pallidipes* to commercial standards of the physiologically-active compounds in zebra skin odor extract. A aldehydes and B ketones. Upper trace is the GC/FID and lower traces represent EAD responses. 1, heptanal; 2, 6-methyl-5-hepten-2-one; 3, octanal; 4, acetophenone; 5, nonanal; 6, decanal; and 7, geranylacetone. The EAD runs were scaled to 10mV/div.
(TIF)
