# Supplementary figures and images for: Zebra skin odor repels the savannah tsetse fly, Glossina pallidipes (Diptera: Glossinidae)
Source: PLoS Negl Trop Dis. 2019 Jun 10;13(6):e0007460. doi: 10.1371/journal.pntd.0007460 (PMC6586361; doi:10.1371/journal.pntd.0007460)

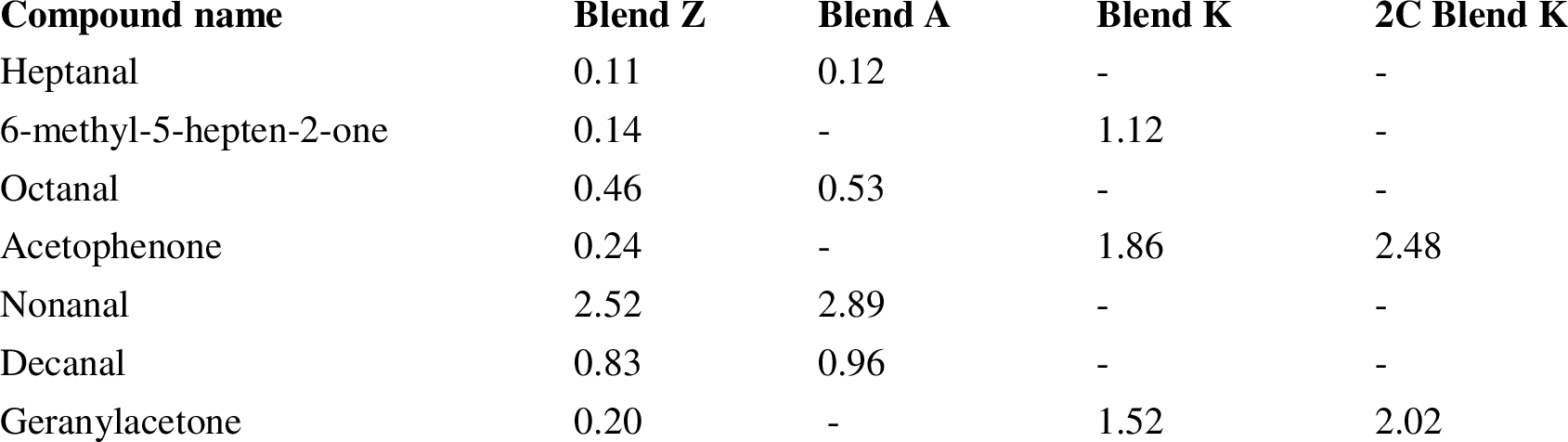

Supplement: S1 Table — (TIF) [file pntd.0007460.s001.tif]

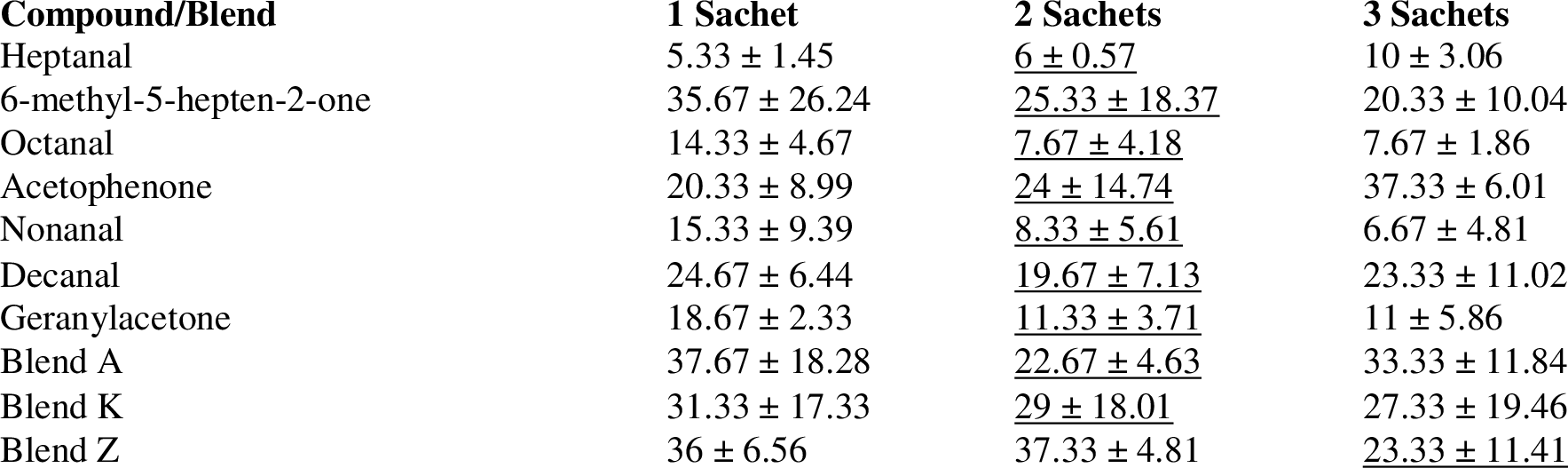

Supplement: S2 Table — Blend A, K, and Z indicate 4-component blend of aldehydes, 3-component blend of ketones and 7-component blend of all EAD-active compounds, respectively, in their natural ratios of occurrence in zebra skin odor. Values underlined represent catches at optimum repellent dose (i.e. dose with the least catch or not significantly different from the dose that did). (TIF) [file pntd.0007460.s002.tif]

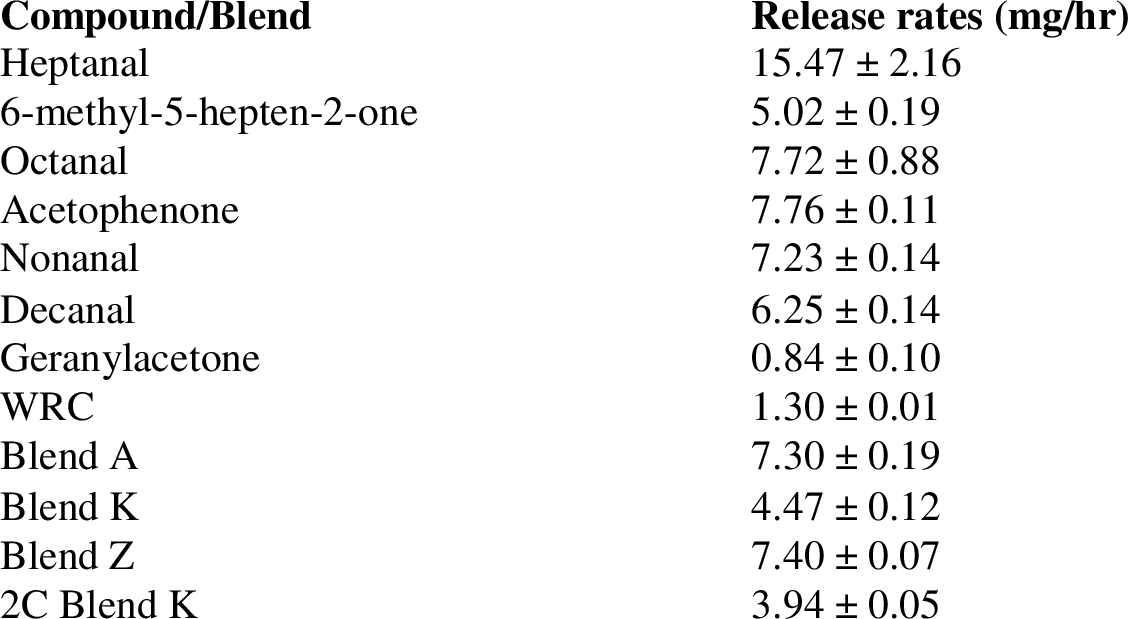

Supplement: S3 Table — Blend A, K, Z and 2C Blend K indicate 4-component blend of aldehydes, 3-component blend of ketones, 7-component blend of all EAD-active compounds and 2-component blend of ketones respectively, in their natural ratios of occurrence in zebra skin odor. (TIF) [file pntd.0007460.s003.tif]

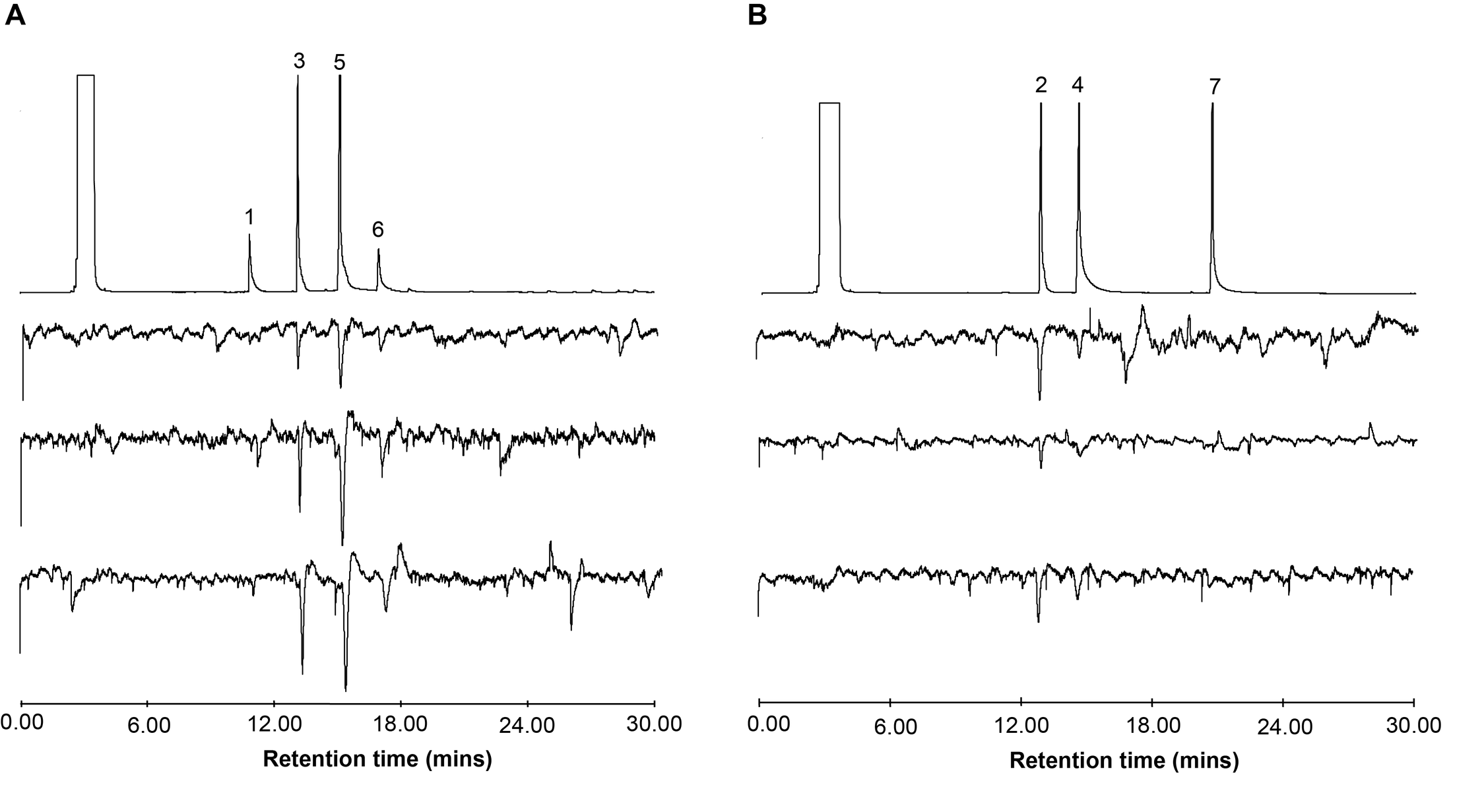

Supplement: S1 Fig — A aldehydes and B ketones. Upper trace is the GC/FID and lower traces represent EAD responses. 1, heptanal; 2, 6-methyl-5-hepten-2-one; 3, octanal; 4, acetophenone; 5, nonanal; 6, decanal; and 7, geranylacetone. The EAD runs were scaled to 10mV/div. (TIF) [file pntd.0007460.s004.tif]
